# Supplementary material for: Stakeholders’ perspectives on Public Health Medicine in South Africa
Source: PLoS One. 2019 Aug 28;14(8):e0221447. doi: 10.1371/journal.pone.0221447 (PMC6713439; doi:10.1371/journal.pone.0221447)
Supplement: S1 Table — (DOCX) [file pone.0221447.s001.docx]

| **Type** | **Number** | **PHM specialists** |
| --- | --- | --- |
| **Academic institutions** | | |
| Trainers of PHM specialists | 4 (13%) | 4 |
| Trainers of other public health professionals | 2 (6%) | 1 |
| **Employers of public health personnel** | | |
| Managers from national government | 2 (6%) | 2 |
| Managers from provincial government in health | 2 (6%) | 2 |
| Managers from health district | 1 (3%) | 0 |
| National Health Laboratory Service | 1 (3%) | 0 |
| Medical Research Council (MRC) | 1 (3%) | 1 |
| Other governmental employers | 2 (6%) | 2 |
| Non-governmental organisations | 1 (3%) | 0 |
| Private sector corporations | 2 (6%) | 1 |
| Consultants to government | 5 (16%) | 4 |
| **Other** | | |
| Retired academics | 6 (19%) | 4 |
| Clinicians | 2 (6%) | 1 |
| **Total** | **31 (100%)** | **22 (71%)** |
