# Supplementary material for: Stakeholders’ perspectives on Public Health Medicine in South Africa
Source: PLoS One. 2019 Aug 28;14(8):e0221447. doi: 10.1371/journal.pone.0221447 (PMC6713439; doi:10.1371/journal.pone.0221447)
Supplement: S1 File — (DOCX) [file pone.0221447.s002.docx]

**Interview guide for *employers* of PH skilled personnel:**

Thank you so much for agreeing to take time out for the interview. As part of my PhD I am doing a study to understand what the opinions of key senior health managers are about public health and skills needed to improve the health status of our population.

1. I want to discuss what you see as the major challenges in the delivery of health services that impact on the health of our population.

2. What do you think are the gaps in expertise?

4. If you were given an increase your budget, what skills and personnel would you prioritise?

5. What do you see as the place of public health in the services?

6. What you understand as the current role of PH trained personnel in the services?

7. Do you have any experience of personnel trained in Public Health? MPH? Dr with MPH? PHM specialist? Can you describe what added value they gave if any?

8. No posts for staff with PH qualifications. Why is that?

9. Are there any areas that you feel public health expertise would be useful/essential?

10. If you were given the option to employ a health professional with an MBA or a MPH which would you chose and why?

11. What roles would the MPH graduate play compared to the PHM specialist?

12. Where do you see the future employment of personnel with public health skills in the services as being?

13. What difficulties do you see in the ability of the services (provincial/district level) to attract or retain professional staff with Public Health skills?

14. What recommendations would you make to training institutions of public health about what competencies they should focus on or change.

15. Do you have any other thoughts and comments that you would like to add?

**2. Interview guide for *trainers* of PH skilled personnel:**

Thank you so much for agreeing to take time out for the interview. As part of my PhD I am doing a study to understand what trainers of public health professionals opinions about public health and skills needed to improve the health status of our population, and how they are contributing to that.

1. I want to first talk about what you see as the major challenges in the delivery of services that impact on the health of our population.

2. What do you think the place of public health is in the public sector health services?

3. Do you think that public health expertise is appreciated by the department of health?

4. What sets of PH skills do you think are the biggest priority for the services?

5. You train post graduates in diplomas/master in public health. What is the intention of the training?

6. What are the jobs that your graduates move into?

7. Historically the PHM or CH specialist was the health manager for a municipality or became a hospital superintendent. This is no longer a requirement. What do you think about this shift?

8. A formal public health qualification is not required for positions in the health sector in South Africa. Why do you think that is so?

9. What impact do you think public health professionals can make in the health sector?

10. What do you think the differences between a PHM specialist and a MPH graduate with and without a MBChB are?

11. For doctors: What do you think the attitude is of the medical profession to public health expertise and PHM specialists? For what sets of expertise?

12. Do you think that PH attracts the calibre of registrars that you think could have the impact you intend? If not why not?

13. What recommendations would you have as to what the services should do about incorporating PH expertise, professionals and specialists into the services?

14. What recommendations would you make about the focus and direction of educational institutions for the training of PH professionals?
